# Insights into coral reef benthic dynamics from nonlinear spatial forecasting

**DOI:** 10.1098/rsif.2019.0047

**Published:** 2019-04-10

**Authors:** Dylan E. McNamara, Nick Cortale, Clinton Edwards, Yoan Eynaud, Stuart A. Sandin

**Affiliations:** 1 Department of Physics and Physical Oceanography, Center for Marine Science, University of North Carolina, 601 South College Road, Wilmington, NC 28403, USA; 2 Center for Marine Biodiversity and Conservation, Scripps Institution of Oceanography, University of California – San Diego, 9500 Gilman Drive, La Jolla, CA 92093-0202, USA

**Keywords:** nonlinear forecasting, coral–algae, determinism

## Abstract

Nonlinear time-series forecasting, or empirical dynamic modelling, has been used extensively in the past two decades as a tool for distinguishing between random temporal behaviour and nonlinear deterministic dynamics. Previous authors have extended nonlinear time-series forecasting to continuous spatial data. Here, we adjust spatial forecasting to handle discrete data and apply the technique to explore the ubiquity of nonlinear determinism in irregular spatial configurations of coral and algal taxa from Palmyra Atoll, a relatively pristine reef in the central Pacific Ocean. We find that the spatial distributions of coral and algal taxa show signs of nonlinear determinism in some locations and that these signals can change through time. We introduce the hypothesis that nonlinear spatial determinism may be a signal of systems in intermediate developmental (i.e. successional) stages, with spatial randomness characterizing early (i.e. recruitment dominated) and late-successional (i.e. ‘climax’ or attractor) phases. Common state-based metrics that sum community response to environmental forcing lack resolution to detect dynamics of (potential) recovery phases; incorporating signal of spatial patterning among sessile taxa holds unique promise to elucidate dynamical characters of complex ecological systems, thereby enhancing study and response efforts.

## 1. Introduction

When scientists take data from a natural system, they do so with the hope that the underlying dynamics, the determinism, will be discovered. If a system is dominated by noise, even a mistake-free scientist will not discover the deterministic signal, as the system's underlying behaviour is masked by a connection of the measured variables to a large number of other degrees of freedom whose dynamics are not known.

Until recently, in experiments where noise is not a problem either through filtering or carefully controlled experimentation, linear methods were the approach of choice for discovering deterministic structure. The limitation of linear methods is the relatively small set of possible system behaviours they are able to elucidate. Linear dynamics can only lead to exponential growth, exponential decay or periodic oscillations, that themselves could also grow or decay; any irregular behaviours in time are attributed to random inputs. Nonlinear time-series analysis is a data-intensive approach that can reveal evidence of a wide range of nonlinear dynamics in the search for deterministic structure [1]. In practice, for many ecological investigations that use nonlinear time-series methods, the goal has been to characterize the extent to which a system is dominated by nonlinear dynamics versus outside forcing from random influences [2,3]. In these efforts, care is taken to ensure the analysis focuses on regions of the data that step beyond auto-correlated behaviour that could result from linear decays of external perturbations. More recent work has used the forecasting capability that results from assuming nonlinear dynamics as a mechanism to predict the future evolution of the system [4].

Similar analyses and interpretations used for nonlinear time-series analysis can be applied to spatial data. Linear methods assume the same simple types of spatial configurations—spatial growth, decay, oscillation. Analogous to interpretations based upon linear models of temporal systems, any spatial configurations that deviate from simple patterns in linear spatial systems must be assumed to result from spatial stochasticity. Nonlinear methods from time-series analysis have been applied to spatial data [5], with a concomitant increase in the types of spatial configurations that are interpretable. However, as with time-series analysis, one must again be careful that the analysis focuses on regions of the spatial data beyond autocorrelations. That said, proper application of nonlinear time-series analysis to spatial data provides the capability of determining whether irregular spatial configurations are nonlinear or random.

If the nonlinear analysis is applied to spatial data at varying points in time, the results hold promise for revealing how the role of nonlinear determinism in the spatial configuration might be evolving. For example, a spatial configuration might change from being nonlinear deterministic to being random, or vice versa, at two moments in time. In such a case, aggregated measures of the spatial configuration such as spatial averages would not reveal the dynamics if the aggregated values have not changed. As a natural system example, much of the focus in coral reef data collection and analysis efforts has been on changes in the percentage of benthic cover of total coral or algal species [6–8]. Point pattern analysis provides a means for distinguishing random versus clustered or regularly distributed patterns; however, this technique is not suitable for categorical data that fill a large domain. Further, point pattern analyses for the entire benthos will not reveal dynamical differences in cases where changes in species borders occur without changes in species centre of mass. Here, we apply nonlinear spatial forecasting to spatially explicit, landscape level (hundreds of square metres) data of coral reef taxa [9] to explore (i) whether evidence of nonlinear spatial determinism is perceptible, and if so, (ii) whether changes in the nonlinear spatial determinism of benthic reef species in the same location are observable through time (year timescale).

## 2. Material and methods

Nonlinear time-series forecasting [1], or empirical dynamic modelling [4], is based on Taken's Theorem [10], which says that a time series of a single measurable variable can be embedded as a trajectory in an *m*-dimensional space by constructing vectors in the space as

2.1 $$z=(x_{n},x_{n-\tau },x_{n-2\tau },\ldots ,x_{n-(m-1)\tau }).$$

Here, *τ* is a temporal lag whose value is sometimes set to the first minimum found in the mutual information between lagged values of the time series [11]. There are systematic ways to choose the dimension, *m*, of the embedding, such as the false near neighbours test [11], but most often the analysis is done over a range of dimensions and then reported for the dimension that provides the most insight. The essence of Taken's Theorem is that a complete picture of the dynamical evolution of the system can be captured with this embedding. Said another way, there is a one-to-one mapping between the phase space attractor of the full system and the reconstructed attractor in the embedded space for the system.

As an analysis technique, the embedding allows one to probe the extent to which a system is nonlinear deterministic, as opposed to being dominated by random noise. Specifically, this is done by splitting the time series into two regimes, a training and a testing set. The training set is used to reconstruct the dynamical evolution of the system in the embedded space. This is achieved using equation (2.1) and creating a series of vector positions in an embedded space that when linked together, reveal trajectories in that space. Next, a point from the testing set is embedded in the same space, and one then probes whether the training set trajectories that are near to the testing point are good forecasters of the future evolution of the testing point. If one finds that the trajectories nearby to the testing point serve as better forecasts than using an average of a large number of trajectories well-separated in space, then the system in question has the hallmarks of nonlinear determinism. In such a case, the dynamical evolution of the system is predicated on a set of initial conditions, and once known, the behaviour of the system follows a set of unique rules captured in the flow of the phase space behaviour.

Exploring determinism in spatial configurations can be done in the same manner [5]. Given an image, a region of the image can be used as the training region, where the locations in the region *x*, *y* are embedded into an (2*m*_*x*_ + 1) by (2*m*_*y*_ + 1) dimensional space with the vector

2.2 $$Z_{i}^{j}=(s_{i-m_{x}\tau }^{\;j-m_{y}\tau },\ldots ,s_{i-m_{x}\tau }^{\;j+m_{y}\tau },\ldots ,s_{i+m_{x}\tau }^{\;j-m_{y}\tau },\ldots ,s_{i+m_{x}\tau }^{\;j+m_{y}\tau }).$$

Once all points in the training region are embedded, a location in the testing region of the image can be chosen to explore nonlinear spatial determinism. This is done by probing how accuracy in spatial forecasts a fixed direction away from the test location depends on the training set's near neighbours used to generate the forecast, where again nearness of neighbours is based on how similar the training vectors are to the test vector. Specifically, if the spatial forecasts degrade as one uses points farther and farther away in the embedded space (less similar vectors), then the spatial configuration is nonlinear deterministic. That is to say, the spatial configuration is an important determinant in how the larger surrounding region is occupied.

Previous work exploring nonlinear spatial determinism with the phase space embedding technique exclusively focused on continuous data [5]. In spatial data collection efforts such as land-use or species distributions, the data are often categorical. The workflow for analysing nonlinear determinism in these cases is essentially the same [12], but care must be taken to make predictions outside regions of a similar discrete value.

## 3. Results

**Figure 1.**
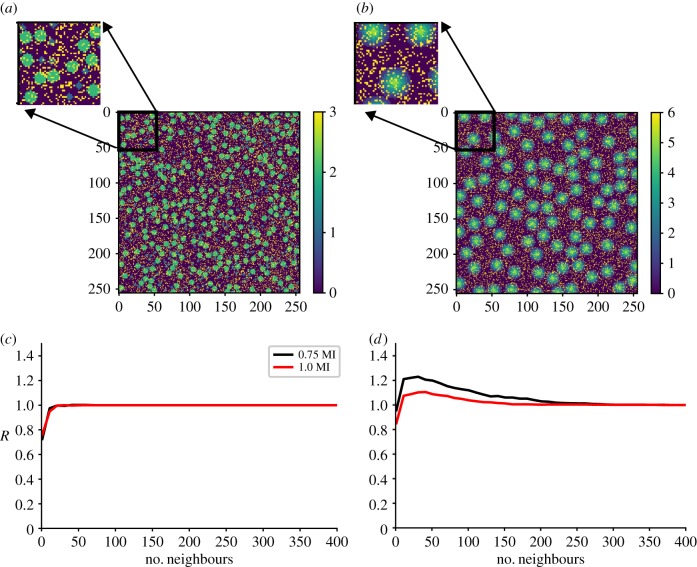
Nonlinear spatial forecasting results from analysis of surrogate spatial data (256 × 256 pixels). (*a*) A random configuration of discrete spatial classes. (*b*) A deterministic arrangement of classes. For both (*a*) and (*b*), the background is class zero. For the random image, classes one and two are larger circles arranged randomly in space and class three are single pixels randomly placed in space. For the deterministic image, class one surrounds class two, which surrounds class three, which surrounds class four, which surrounds class five and again class six is single pixels randomly placed in space. Graphs (*c,d*) show the forecasting skill for the images in (*a,b*), respectively, relative to a mode forecast, *R*, measured against the number of near neighbours used to make the forecast for forecast distances 0.75 to the minimum in mutual information distances (black) and 1.0 to the minimum in mutual information distances (red) away in space. (Online version in colour.)

As an illustration of nonlinear forecasting in space for categorical data, we have first constructed two simple spatial images with discrete values at each pixel. The first image is of circles of two different sizes placed randomly over a two-dimensional grid (figure 1*a*). The second is a series of four concentric circles scattered across the domain (figure 1*b*). For both of the images, we have introduced random noise by placing an additional class in individual pixel locations at random locations throughout the image. For context, these two images can be thought of as a domain with a backdrop of high spatial frequency randomness, where in one case, two different sized circle regions of given types are randomly distributed in space, while in the other, the regions of given type are always surrounded in space by a second type, and those are always surrounded by a third type, and those surrounded by a fourth type. The former is clearly random while the latter is deterministic in the rules that dictate surrounding neighbours.

For each image, a subset (we chose 70%) of the domain is taken as the training portion for nonlinear spatial forecasting, with the rest used as the testing portion. Vectors are constructed in an embedded space according to equation (2.2) where we have chosen the lag as one pixel and the embedding dimension using the first minimum in the mutual information, which for our spatially discrete data set we found according to

3.1 $$I(\Delta )=\underset{s_{n},s_{n+\Delta }}{\sum }P(s_{n},s_{n+\Delta })\log\left[\frac{P(s_{n},s_{n+\Delta })}{P(s_{n})P(s_{n+\Delta })}\right].$$

Here $s_{n}\;$ refers to a given pixel and $s_{n+\Delta }$ refers to a pixel some lagged distance away in either a row or column of the data. We performed the calculation of equation (3.1) using only rows and using only columns, and we did so for a randomly chosen 20% of the rows and columns in the spatial data. The embedding dimension we used to construct equation (2.2) was the mean across the 20% of rows and columns we analysed of the minimum in the mutual information curves from equation (3.1). For the images shown in figure 1, this corresponds to five and seven pixels for the random and deterministic patterns, respectively. We will refer to an embedded point as a placket, since the construction of a vector according to equation (2.2) with a lag of one pixel, makes a small square placket within the image. In this case, the plackets are boxes that are 5 × 5 pixels and 7 × 7 pixels, respectively, with each centred on the pixel in question. The training set is populated by repeated calculation of vector values for all plackets within the training region. Next, a location in the testing region is chosen at random and a placket is constructed in the same manner, and the entire test region of plackets is searched for the nearest matching placket. In the case of our discrete data, the distance in the embedded space between a test placket and training placket is simply the sum of the number of pixels that do not match in the placket, and thus the nearest neighbour is the one with the smallest number of different pixels when comparing embedded vectors (equation (2.2)).

A forecast of a test placket's surrounding region is made using near neighbours to the placket in question, where a neighbour is defined with respect to the embedded space. Consider a forecast made 10 pixels away from a test placket using only the closest near neighbour for generating the forecast. In this case, the forecast 10 pixels away from the test placket would be the pixel value that is 10 pixels away from the placket in the training region that most closely matches the placket from the test region. Forecasts 10 pixels away using say, the three closest near neighbours, are generated by finding the three plackets that are the most similar to the test placket. The forecast 10 pixels away from the test placket in this case is made by taking the mode of the three pixels that are 10 pixels away from three nearest neighbour plackets. In the work reported here, the predictions are made in the upwards direction away from the pixel in the centre of the placket but we also flip the image in all four directions in our analysis so our predictions are isotropic. Previous work analysing continuous spatial data used a range of different ways to combine the evolutions of neighbours in the embedded space to make a forecast from a test point. Early efforts used forecasts based on simple averages of neighbour trajectories. Following that, weighted averages of neighbours were more commonly chosen, with the weights assigned based on distance to the neighbour in the embedded space. More recent analyses have incorporated simplex projections of points surrounding the test point [2]. In our case, with discrete data, taking the mode of the neighbour forecasts seems fitting in capturing the basic concept of averaging the trajectories.

Figure 1*c,d* shows the forecast skill as a function of the number of neighbours used to make forecasts, where the neighbours are chosen in order of how similar their embedded vectors are to the test vector. The forecast skill, *R*, is measured as the ratio of the number of correctly forecast pixels to the number of pixels forecast correctly if the forecast was the mode of the data. Forecast skill shown at a given value of neighbours comes from forecasting half of the test points from the testing portion of the domain (about 16 000 points in this case). With this simple ratio, values of *R* larger than 1.0 show that the forecast is providing an improvement relative to predicting the mode. To systematically make sure that forecasts are always made into regions with new information relative to the centre pixel of a given placket, we only show forecast skill for forecasts far enough away in space that the distance is near the minimum in the mutual information (which is a distance of five and seven pixels for the random and deterministic images). When making forecasts out to 75% and 100% of the distance that represents the minimum in mutual information for the data, the hallmarks of determinism are clearly visible. For the case of random circles, there is no benefit to using only near neighbours in the embedding space (figure 1*c*), which is to say a given placket configuration offers no utility in knowing what surrounds the region in space. Conversely, with circles of a given type always surrounded by circles of the same type (albeit, even with noise scattered about), the best forecasts of the surrounding region come from considering other regions in the domain that are most similar (figure 1*d*). In other words, the spatial configuration, as is definitional from the manner in which we made the data, is deterministic.

**Figure 2.**
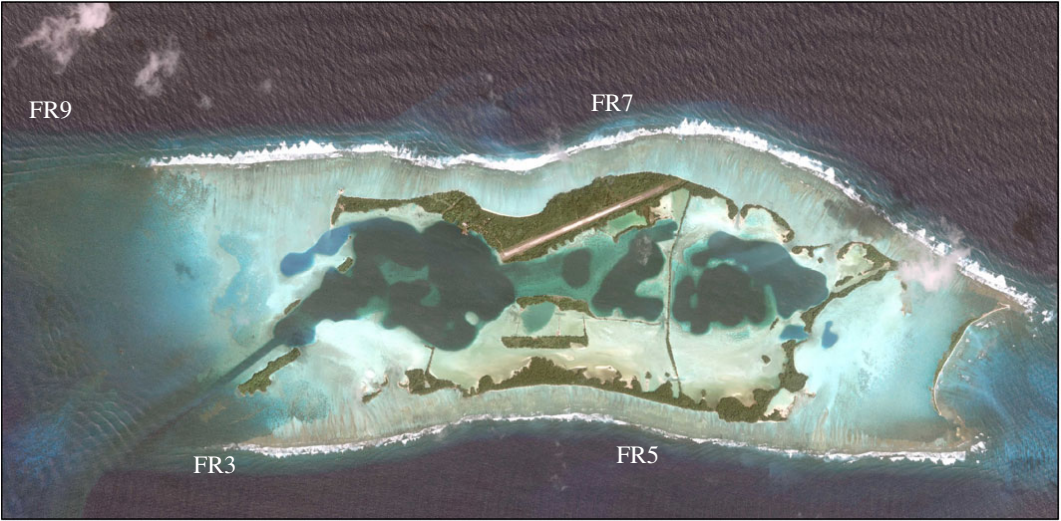
Aerial view of Palmyra Atoll in the northern Line Islands showing the four regions, FR3, FR5, FR7 and FR9 where photomosaic images of the reef benthos were acquired for analysis. (Online version in colour.)

To explore nonlinear spatial determinism in a natural system, we consider spatial data from large-scale photographic images that have been collected from Palmyra Atoll (figure 2), a US Fish and Wildlife National Wildlife Refuge located approximately 1600 km south of Oahu, HI. The coral reef in Palmyra has remained essentially free of human disturbance for the past 50 years [13]. The field effort to collect the 100 m^2^ photomosaics has been described previously [9]. Briefly, a diver equipped with two mounted cameras swims about 1.5 m above the reef in a gridded pattern, with the cameras taking pictures every second. The resulting series of images are stitched together with an image processing algorithm to form one large, orthorectified image of the reef. Photomosaics are then post-processed for identification of individual species within the image. This is done using the operational definition of a colony as a contiguous patch of live tissue and having a human operator designate every coral or algal patch within an image to the finest taxonomic level possible (principally to the level of genus or species, based upon morphometric distinctiveness). For this study, the photomosaics are categorized at the species level according to table 1 with the particular species list shown taken from previous work on the photomosaics [9].

**Table 1.** Species ID and name for the classified photomosaic shown in figure 3.

ID	species	ID	species	ID	species
1	*Acropora* (branching)	17	*Halimeda*	33	*Porites* (massive)
2	*Acropora* (corymbose)	18	*Hydnophora exesa*	34	no species
3	*Acropora* (plating)	19	*Hydnophora microconos*	35	*Porites superfusa*
4	*Astreopora myriophthalma*	20	*Leptastrea*	36	*Psammocora*
5	no species	21	*Leptoseris*	37	*Sarcophyton*
6	*Clavularia*	22	*Lobophyllia*	38	soft coral
7	corallimorph	23	no species	39	*Stylophora pistillata*
8	*Dictyosphaeria*	24	*Montastrea curta*	40	*Turbinaria reniformis*
9	no species	25	*Montipora* (encrusting)	41	unknown
10	*Favia matthai*	26	*Montipora* (plating)	42	unknown (encrusting)
11	*Favia stelligera*	27	other	43	unknown (massive)
12	*Favites* (encrusting)	28	*Pavona* (submassive)	44	unknown (submassive)
13	*Favites* (submassive)	29	*Pavona varians*	45	zooanthid
14	*Fungia* (multiple polyp)	30	*Platygyra*	0	unidentified
15	*Fungia*	31	*Pocillopora*		
16	no species	32	*Pocillopora eydouxi*		

**Figure 3.**
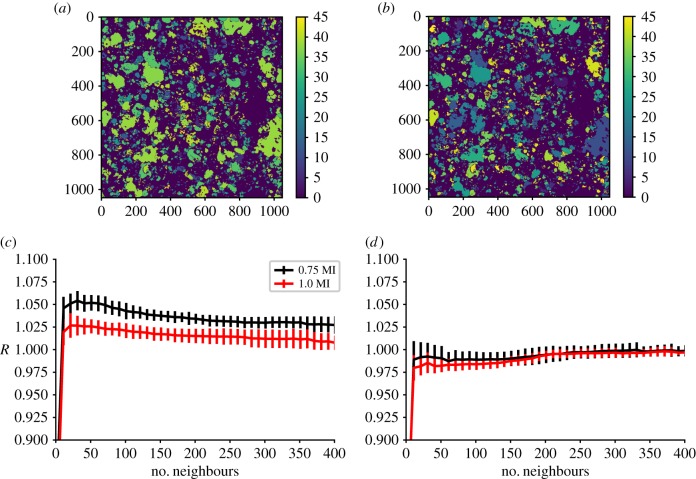
Nonlinear spatial forecasting results from analysis of the coral benthos at FR7 and for an image with the species randomly arranged for the site FR7. (*a*) The coral benthos at FR7 with species assigned by number. (*b*) The same spatial arrangement as (*a*) but with species randomly numbered. Graphs (*c,d*) show the forecasting skill for the images in (*a,b*), respectively, relative to a mode forecast, *R*, measured against the number of near neighbours used to make the forecast for forecast distances 0.75 to the minimum in mutual information distances (black) and 1.0 to the minimum in mutual information distances (red) away in space. Vertical error bars represent one standard deviation from the mean when doing the analysis using varying regions for testing and training. (Online version in colour.)

Nonlinear spatial forecasting suggests that the spatial configurations of coral and algal taxa from location FR7 at Palmyra in 2012 show signs of nonlinear determinism. Figure 3 shows the forecast skill, *R*, as a function of neighbours used to make the forecast for raw orthorectified images where each species has been given a unique identification number (figure 3*a*) compared to the case where the identification numbers have been assigned randomly to every contiguous species region (figure 3*b*). The latter represents surrogate data that serve as a baseline test, whereby the spatial configuration is made to be random. In this analysis, the placket size was set to 20 × 20 pixels, as a lag of 20 pixels was near the minimum in the mutual information (equation (3.1)). Furthermore, the placket comparisons are based on the similarity of pixel class, in this case, species ID (table 1) within the placket. For the surrogate data, the analysis shows that forecast skill is not enhanced by forecasts generated from regions similar to a region in question (figure 3*d*). In fact, at best the forecast skill approaches a forecast of the mode of the data. For the authentic mosaic, forecast improvement over the mode is found by using only those plackets close in the embedded phase space (figure 3*c*). For each of the images, the analysis has been done by choosing different subsets of the domain as the training and testing plackets. Therefore, we are operating under the assumption of spatial isotropy and the error bars reflect the standard deviation over the various different trials of training and testing choice.

**Figure 4.**
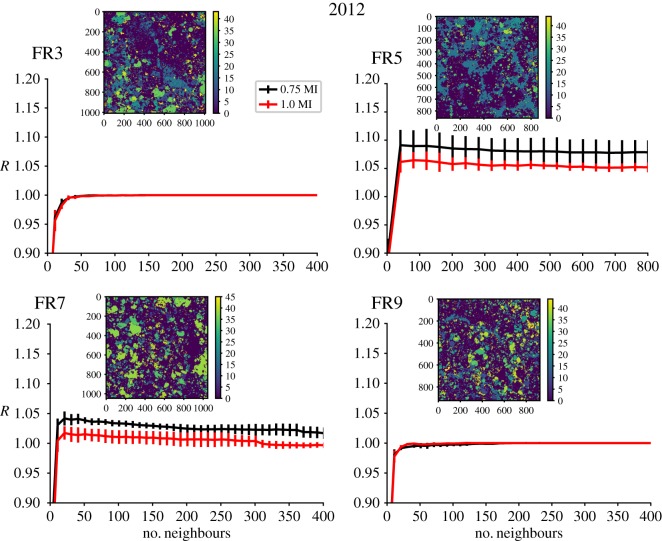
Nonlinear spatial forecasting results from analysis of the coral benthos at the four locations in Palmyra from figure 2 during the year 2012 with mosaic images of the benthos inset within each plot. The plots show the forecasting skill relative to a mode forecast, *R*, measured against the number of near neighbours used to make the forecast for forecast distances 0.75 to the minimum in mutual information distances (black) and 1.0 to the minimum in mutual information distances (red) away in space. Vertical error bars represent 1 s.d. from the mean when doing the analysis using varying regions for testing and training. (Online version in colour.)

**Figure 5.**
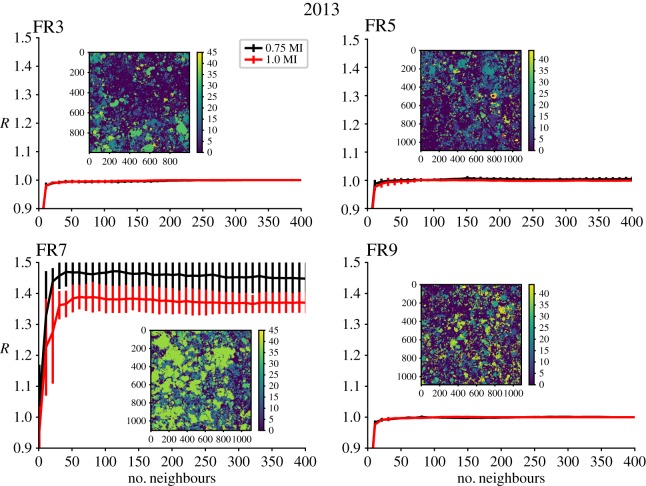
Nonlinear spatial forecasting results from the analysis of the coral benthos at the four locations in Palmyra from figure 2 during the year 2013 with mosaic images of the benthos inset within each plot. The plots show the forecasting skill relative to a mode forecast, *R*, measured against the number of near neighbours used to make the forecast for forecast distances 0.75 to the minimum in mutual information distances (black) and 1.0 to the minimum in mutual information distances (red) away in space. Vertical error bars represent one standard deviation from the mean when doing the analysis using varying regions for testing and training. (Online version in colour.)

When the nonlinear spatial analysis is applied to orthorectified images of four of the same sites in both 2012 and a year later in 2013, the signal of nonlinear determinism, that forecast skill is improved using plackets most similar to a region, remains for one site (FR7) but is absent for the remaining three sites (figures 4 and 5). For FR7 in 2013, the overall forecasting capability has improved over the shown range of near neighbours but importantly the best forecasts still occur at lower numbers of neighbours (and this peak was enhanced when probing further into near neighbours). The reason for the overall improvement is that a mode forecast, which is the baseline for comparison in our scoring metric, performs worse in 2013 at FR7. At both FR3 and FR9, the spatial configurations of benthic taxa appear random in both 2012 and 2013. FR5 shows some improvement in spatial forecasts using only similar near neighbours and hence indicates nonlinear deterministic spatial structure in 2012, but signs of determinism are lacking in the 2013 mosaic as forecasting improvement over the mode prediction is not found.

## 4. Discussion and Conclusion

When assessing change on reefs, framework-building corals are commonly grouped together to provide a single metric of per cent coral cover. While useful for providing broad comparisons across locations or through time [14,15], as an aggregate measure, per cent cover overlooks important changes in coral community structure. Consider two extreme cases of a reef showing no change in per cent cover of coral between two time points. In one case, the evidence of no change may reflect stasis in the coral assemblage structure—no change in the abundance, diversity or size structure of the constituent corals. In the second case, there may be complete mortality of all corals of one species with associated recruitment and growth of a different species. Both cases result in comparable per cent coral cover but the dynamical structure of these two reefs is quite distinct, one reflecting stability and the latter reflecting demographic dynamism. The major reef-building corals are colonial and clonal, and thus feature population dynamics that are more complex than the classic life/death processes of solitary organisms [16]. In particular, corals can respond to stress events with the capacity for shrinking and propagation via fragmentation and avoid entire organism mortality with this partial mortality [17,18]. Furthermore, the extensive regenerative capabilities of corals can enable rapid reoccupation of space following disturbance events or cessation of stressful conditions [19–21]. As a result, coral growth strategies can reinforce stability by providing the deterministic dynamics for rapid recovery from disturbances.

The coral communities at Palmyra show evidence of dynamical evolution when their spatial configuration changes from showing signs of nonlinear deterministic structure to being randomly arranged; this appears to be the case for the community surveyed at FR5. A possible interpretation of this evolution is variation in the disturbance regimes of the study sites prior to 2012. This interpretation relies on the dynamical characteristic of strong nonlinear determinism when a system is following a phase space trajectory that is moving toward an attractor, having been recently perturbed from its attractor state [22]. When considering the behaviour of a model coral reef system across two-dimensional patterns of clustering, Brito-Millán and colleagues [22] found a characteristic signal of nonlinear determinism only during the so-called transient phase, namely the period of system organization before reaching the final dynamical attractor. Large or frequent disturbance events would take the system from its attractor, and as the system is then moving back into the attractor configuration over a longer time period, strong nonlinear system dynamics would guide the system back to its stable attractor state. In such a scenario, nonlinear determinism would be more evident following the disturbance than when back in the attractor. For sites that remain in a random benthic configuration, FR3 and FR9, the same interpretation would suggest that these sites were not disturbed from their attractors during the study period. At FR7, which maintained signs of nonlinear determinism in 2012 and 2013, the disturbance interpretation would suggest that this site is in a constant state of reaction to large events.

The environmental context for these sites loosely agrees with these interpretations [23–25]. Large energy SW wave events occur infrequently at FR5. For location FR7, large winter NW wave events occur often and directly impact the study site. The regions of little evidence for disturbance, FR3 and FR9, are situated in regions of the island that receive somewhat less direct wave energy. Importantly, these sites have dramatically higher abundances of several key taxa (*Fungia* sp., *Montipora* sp. and *Porites superfusa)* which have been shown to be locally clustered. Additionally, the spatial patterns of these groups largely fit models of biotic clustering which we hypothesize to be driven by partial mortality and fragmentation [9,26], both which require external perturbation (e.g. storms, bleaching, predator/disease outbreak) to occur. As these processes also result in higher numbers of individuals, the large population sizes of these groups at FR3 and FR9 might reflect success in these groups following historical disturbance. Furthermore, the total abundance and per cent cover of all taxa combined are nearly twice as high at FR3 and FR9. Combined with the dominance of fast-growing taxa at these sites, the resulting pre-emption of space might prevent the communities at these sites from moving sufficiently far from the attractor for nonlinear dynamics to emerge.

We propose a working model for nonlinear spatial forecasting as a tool for probing ecological system dynamics [27]. At early stages of succession, or immediately following a disturbance event that has reset the spatial configuration, the spatial configuration will be random. Once system dynamics begin to take hold in successional spatial interactions, the spatial configuration shows signs of nonlinear deterministic structure. Eventually, once the system has reached late stages of succession, the strong spatial interactions have dissipated and the spatial configuration again shows signs of randomness. This working model for interpreting spatial nonlinear forecasting at various times agrees with recent modelling work that explored benthic dynamics in coral reefs [22] and it parallels the idea that increasing signs of nonlinearity are harbingers of a system moving between dynamical attractors [28]. As such, spatial forecasting holds promise as a tool for exploring ecological succession and with the recent advances in observing underwater ecological communities, scientists can use the tool to probe a wide array of systems in their response to natural and anthropogenic disturbance.

## Data accessibility

Python code for the spatial forecasting is available at https://github.com/NickC1/skedm. Photomosaics are available within electronic supplementary material in previous work [9].

## References

[1] Kantz H, Schreiber T 2004 Nonlinear time series analysis. Cambridge, UK: Cambridge University Press.

[2] Sugihara G, May RM 1990 Nonlinear forecasting as a way of distinguishing chaos from measurement error in time series. Nature 344, 734 (10.1038/344734a0) 2330029

[3] Hsieh CH, Glaser SM, Lucas AJ, Sugihara G 2005 Distinguishing random environmental fluctuations from ecological catastrophes for the North Pacific Ocean. Nature 435, 336 (10.1038/nature03553) 15902256

[4] Perretti CT, Munch SB, Sugihara G 2013 Model-free forecasting outperforms the correct mechanistic model for simulated and experimental data. Proc. Natl Acad. Sci. USA 110, 5253–5257. (10.1073/pnas.1216076110) 23440207 PMC3612644

[5] Rubin DM 1992 Use of forecasting signatures to help distinguish periodicity, randomness, and chaos in ripples and other spatial patterns. Chaos 2, 525–535. (10.1063/1.165894) 12780001

[6] Mumby PJ, Hastings A, Edwards HJ 2007 Thresholds and the resilience of Caribbean coral reefs. Nature 450, 98 (10.1038/nature06252) 17972885

[7] Bellwood DR, Hughes TP, Folke C, Nyström M 2004 Confronting the coral reef crisis. Nature 429, 827 (10.1038/nature02691) 15215854

[8] Hughes TP 1994 Catastrophes, phase shifts, and large-scale degradation of a Caribbean coral reef. Science 265, 1547–1551. (10.1126/science.265.5178.1547) 17801530

[9] Edwards CB, Eynaud Y, Williams GJ, Pedersen NE, Zgliczynski BJ, Gleason AC, Smith JE, Sandin SA 2017 Large-area imaging reveals biologically driven non-random spatial patterns of corals at a remote reef Coral Reefs 36, 1291–1305. (10.1007/s00338-017-1624-3)

[10] Takens F 1981 Detecting strange attractors in turbulence. In Dynamical systems and turbulence, Warwick 1980 (eds A Dold, B Eckman), pp. 366–381. Berlin, Germany: Springer.

[11] Abarbanel HD, Brown R, Sidorowich JJ, Tsimring LS 1993 The analysis of observed chaotic data in physical systems. Rev. Mod. Phys. 65, 1331 (10.1103/RevModPhys.65.1331)

[12] Cortale N, McNamara D 2017 skedm: Empirical dynamic modeling. J. Open Source Softw. 2, 207 (10.21105/joss.00207)

[13] Sandin SA et al. 2008 Baselines and degradation of coral reefs in the northern Line Islands. PLoS ONE 3, e1548 (10.1371/journal.pone.0001548) 18301734 PMC2244711

[14] Sweatman H, Delean S, Syms C 2011 Assessing loss of coral cover on Australia's Great Barrier Reef over two decades, with implications for longer-term trends. Coral Reefs 30, 521–531. (10.1007/s00338-010-0715-1)

[15] Smith JE et al. 2016 Re-evaluating the health of coral reef communities: baselines and evidence for human impacts across the central Pacific. Proc. R. Soc. B 283, 20151985 (10.1098/rspb.2015.1985) PMC4721084 26740615

[16] Jackson JB, Coates AG 1986 Life cycles and evolution of clonal (modular) animals. Phil. Trans. R. Soc. Lond. B 313, 7–22. (10.1098/rstb.1986.0022)

[17] Highsmith RC 1982 Reproduction by fragmentation in corals. Mar. Ecol. Progr. Ser. 15, 207–226. (10.3354/meps007207)

[18] Hughes TP, Ayre D, Connell JH 1992 The evolutionary ecology of corals. Trends Ecol. Evol. 7, 292–295. (10.1016/0169-5347(92)90225-Z) 21236037

[19] Mackie GO 1986 From aggregates to integrates: physiological aspects of modularity in colonial animals. Phil. Trans. R. Soc. Lond. B 313, 175–196. (10.1098/rstb.1986.0032)

[20] Diaz-Pulido G et al. 2009 Doom and boom on a resilient reef: climate change, algal overgrowth and coral recovery. PLoS ONE 4, e5239 (10.1371/journal.pone.0005239) 19384423 PMC2668766

[21] Gilmour JP, Smith LD, Heyward AJ, Baird AH, Pratchett MS 2013 Recovery of an isolated coral reef system following severe disturbance. Science 340, 69–71. (10.1126/science.1232310) 23559247

[22] Brito-Millán M, Werner BT, Sandin SA, McNamara DE 2019 Influence of aggregation on benthic coral reef spatio-temporal dynamics. R. Soc. open sci. 6, 181703 (10.1098/rsos.181703) 30891282 PMC6408412

[23] Williams GJ, Smith JE, Conklin EJ, Gove JM, Sala E, Sandin SA 2013 Benthic communities at two remote Pacific coral reefs: effects of reef habitat, depth, and wave energy gradients on spatial patterns. PeerJ 1, e81 (10.7717/peerj.81) 23734341 PMC3669270

[24] Williams GJ, Gove JM, Eynaud Y, Zgliczynski BJ, Sandin SA 2015 Local human impacts decouple natural biophysical relationships on Pacific coral reefs. Ecography 38, 751–761. (10.1111/ecog.01353)

[25] Rogers JS, Monismith SG, Koweek DA, Dunbar RB 2016 Wave dynamics of a Pacific Atoll with high frictional effects. J. Geophys. Res. Oceans 121, 350–367. (10.1002/2015JC011170)

[26] Furby KA, Smith JE, Sandin SA 2017 *Porites superfusa* mortality and recovery from a bleaching event at Palmyra Atoll, USA. PeerJ 5, e3204 (10.7717/peerj.3204) 28480135 PMC5417065

[27] Odum EP 1966 The strategy of ecosystem development. Science 164, 262–270. (10.1126/science.164.3877.262) 5776636

[28] Dakos V, Glaser SM, Hsieh CH, Sugihara G 2017 Elevated nonlinearity as an indicator of shifts in the dynamics of populations under stress. J. R. Soc. Interface 14, 20160845 (10.1098/rsif.2016.0845) 28250096 PMC5378125

